# Initial report of percutaneous left atrial appendage closure in hemodialysis patients with atrial fibrillation and high risk of bleeding in Japan

**DOI:** 10.1007/s12928-022-00904-9

**Published:** 2022-12-23

**Authors:** Hiroshi Ueno, Teruhiko Imamura, Shuhei Tanaka, Ryuichi Ushijima, Nobuyuki Fukuda, Koichiro Kinugawa

**Affiliations:** The Second Department of Internal Medicine, University of Toyama, 2630 Sugitani, Toyama, 930-0194 Japan

**Keywords:** Atrial fibrillation, Anticoagulation, Thrombosis

## Abstract

In the countries like Japan where anticoagulation is not recommended in hemodialysis patients, the feasibility of percutaneous left atrial appendage closure (LAAC) in hemodialysis patients with non-valvular atrial fibrillation (NVAF) accompanying high risks of thromboembolic stroke and bleeding remains unknown. Peri-procedural and 45-day clinical outcomes following LAAC using WATCHMAN system, which were performed in our institute between Jun 2020 and April 2022 according to the Japanese Circulation Society guidelines, were retrospectively compared between those with and without hemodialysis. 118 patients (median 79 years, 81 men) consisting of 25 hemodialysis patients and 93 non-hemodialysis patients were included. CHADS score was 3 (2, 4) in the hemodialysis patients and 3 (2, 4) in the non-hemodialysis patients (*p* = 0.98). HAS-BREAD score was 4 (3, 5) in the hemodialysis patients and 3 (2, 3) in the non-hemodialysis patients (*p* < 0.001). All procedures were successful, except for a non-hemodialysis patient with a larger left atrial appendage. There were no major complications during index hospitalization and 45-day observational period, except for a hemodialysis patient with suspected bleeding and a non-hemodialysis patient who died due to cardiac amyloidosis. LAAC seems to be feasible in hemodialysis patients with high risks of thromboembolic events and bleedings.

## Background

Non-valvular atrial fibrillation (NVAF) is the most common cardiac arrhythmia. The prevalence of NVAF has increased with the aging of the world-wide population, affecting approximately 10% in Western countries [1] and approximately 4% in Japan [2]. One of the major and critical complications of NVAF is ischemic stroke. The existence of NVAF increases the risk of ischemic stroke approximately 4–5 times [3]. Current guidelines in Europe, United States, and Japan, recommend anticoagulation therapy using either warfarin or direct oral anticoagulants (DOACs) to prevent thromboembolic ischemic stroke in patients with NVAF and its high risk, according to several risk stratifying scoring systems [4–6].

The strategy for those with end-stage renal disease is controversial. The incidence of NVAF and its impact on increasing the risk of stroke are higher in patients with end-stage renal disease compared with general population [7]. Nevertheless, Japanese guidelines do not allow any anticoagulants for such a purpose among this cohort thus far [6], particularly given a high bleeding risk during anticoagulation therapy [8].

**Fig. 1:**
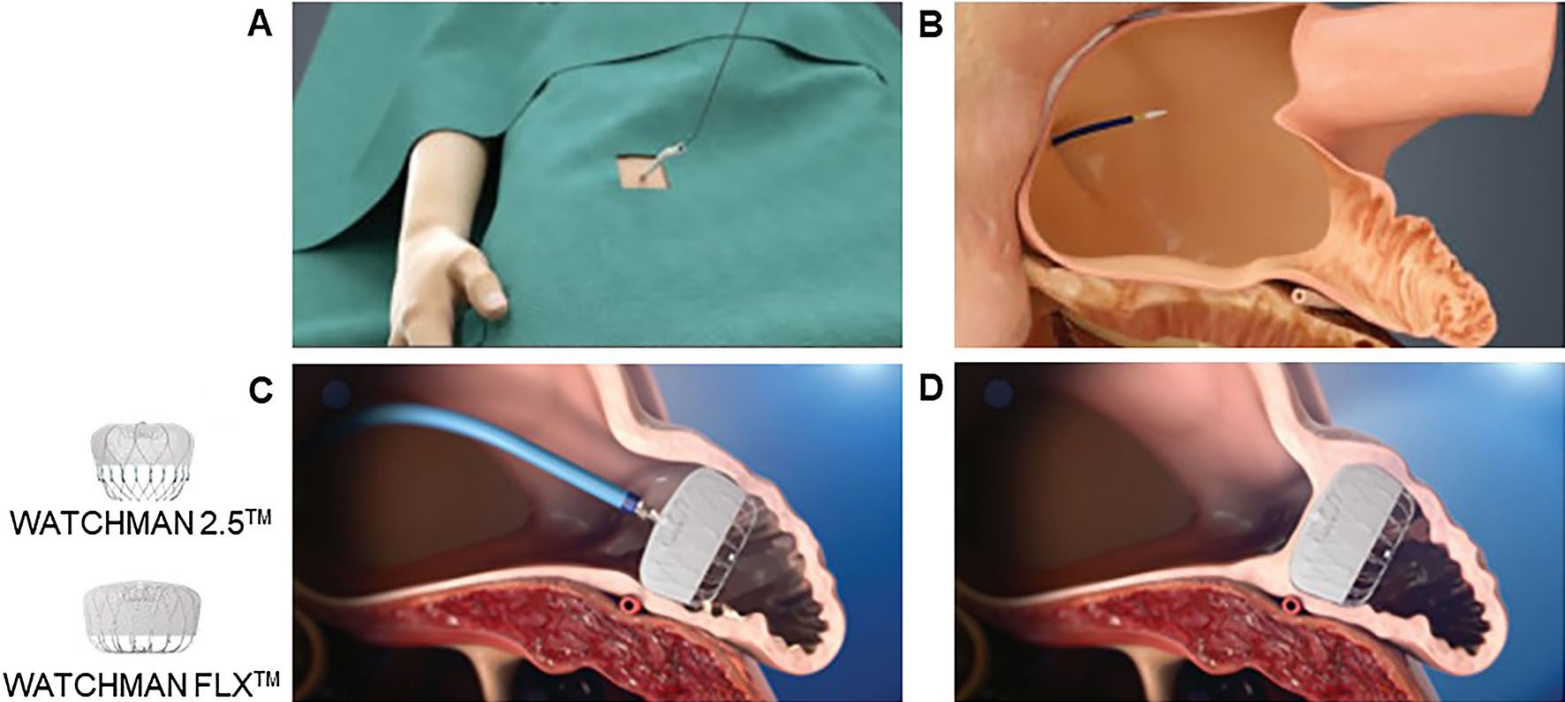
Left atrial appendage closure using WATCHMAN system. A catheter is inserted using a standard percutaneous technique from the femoral vein (**A**). The interatrial septum is crossed using a standard trans-septal access system (**B**). WATCHMAN is deployed and released in the left atrial appendage (**C**). Heart tissue grows over the WATCHMAN implant and the left atrial appendage is permanently sealed (**D**)

Percutaneous left atrial appendage closure (LAAC) is an established therapy for preventing thromboembolic stroke in patients with NVAF, who are not good candidates for long-term anticoagulation therapy (Fig. 1) [9]. However, the detailed clinical implication of LAAC therapy in hemodialytic patients remains uninvestigated [10]. Recently, the safety and efficacy of LAAC for Japanese patients were confirmed in a multi-center single-arm observational study [11], whereas clinical outcomes following LAAC in hemodialysis patients remain uncertain. In this proof-of-concept study, we investigated short-term clinical outcomes of LAAC therapy in NVAF patients with and without hemodialysis.

## Methods

### Patient selection

NVAF was defined as AF without moderate or severe mitral stenosis or mechanical prosthetic heart valve. Consecutive patients with NVAF who received LAAC using WATCHMAN system (Boston Scientific, St. Paul, Minnesota; Fig. 1) in our institute between June 2020 and April 2022 were prospectively included in our institutional registry and this study was retrospectively conducted using this registry data.

According to our institutional protocol, WATCHMAN 2.5 was used between June 2020 and June 2021. WATCHMAN FLX was used from July 2021. The study was approved by the local ethical board (R2020077) and informed consents were obtained from all participants before inclusion.

### Procedure indication

The indication of LAAC was according to the Japanese Circulation Society guidelines. Patients should be those with NVAF who were at high risk of systemic embolisms and were highly recommended to receive anticoagulation therapy according to CHADS_2_ score and CHADS_2_-VASc score. On the contrary, they should also be at high risk of bleeding and satisfy either of them: HAS-BLED score equal to or above 3 points; multiple histories of trauma due to falling; cerebral amyloid angiopathy; requirement of multiple antiplatelets; histories of major bleeding with BARC type 3–5. All patients received transesophageal echocardiography for the anatomical assessments. The final indication was determined by the institutional heart-valve team conference.

The indication of LAAC for the hemodialysis patients was similar to the non-hemodialysis patients. All hemodialysis patients satisfied “A” in the HAS-BLEAD score (i.e., abnormal renal function).

### Procedure

The WATCHMAN2.5 and FLX are self-expanding, nitinol-framed structures ranging in diameter from 21 to 33 mm (WATCHMAN2.5) and from 20 to 35 mm (WATCHMAN FLX), respectively, to accommodate varying LAA anatomy and size. These devices are fixed by anchor at the LAA ostium to avoid embolization and to prevent blood flow in the LAA. LAAC was performed under general anesthesia according to the standard procedure by the board-certified interventionists using angiography, transesophageal echocardiography, and double curve sheath via a trans-septal puncture approach.

### Post-procedure follow-up

**Fig. 2:**
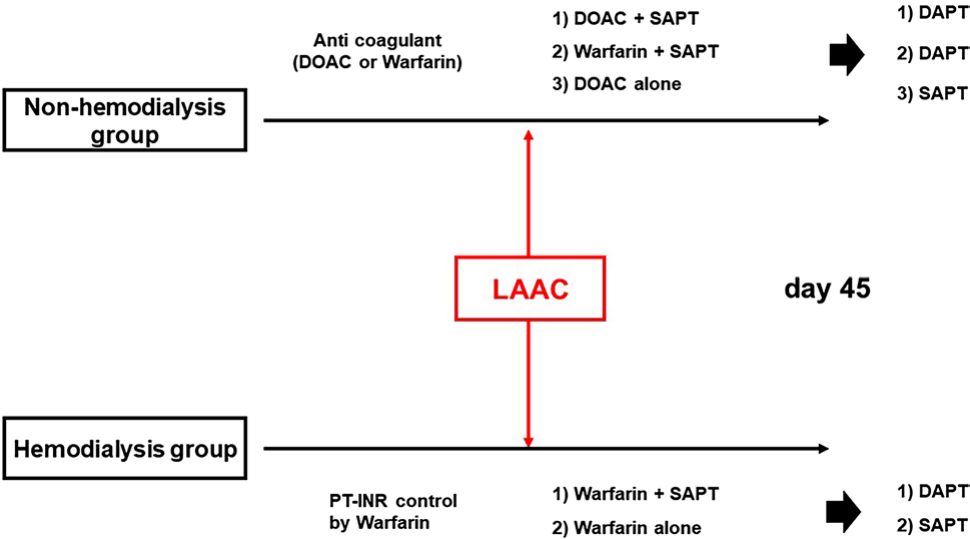
Post-implant drug regimen. *DOAC* direct oral anticoagulation; *SAPT* single antiplatelet using aspirin 100 mg or clopidogrel 75 mg; PT-INR, prothrombin time with international normalized ratio; *LAAC* left atrial appendage closure

Following the procedures, anticoagulation therapy using warfarin or DOAC as well as antiplatelets were continued for 45 days to allow time for device endothelialization: (1) single antiplatelet and DOAC/warfarin with the therapeutic international normalized ratio between 2.0 and 2.6 for those with non-hemodialysis; single antiplatelet and warfarin with a therapeutic international normalized ratio between 1.5 and 2.0 for hemodialytic patients (Fig. 2).

In hemodialysis patients, warfarin and single antiplatelet were converted to dual antiplatelet, and warfarin alone was converted to single antiplatelet following a 45-day follow-up. In non-hemodialysis patients, DOAC and single antiplatelet were converted to dual antiplatelet, warfarin and single antiplatelet were converted to dual antiplatelet, and DOAC alone was converted to single antiplatelet following 45-day follow-up. All medications were converted to single antiplatelet following 6-month follow-up.

### Study outcomes

**Fig. 3:**
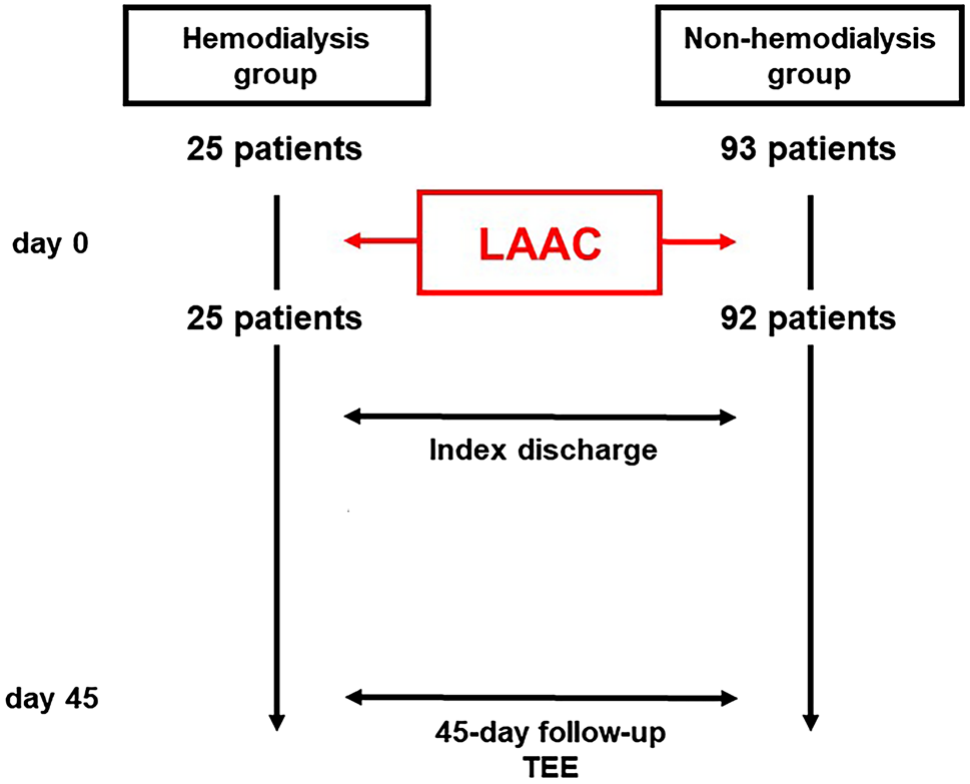
Flow chart from left atrial appendage closure to 45-day follow-up in patients with and without hemodialysis. LAAC was failed due to an inappropriately larger left appendage in a patient in the non-hemodialysis group. *LAAC* left atrial appendage closure; *TEE* transesophageal echocardiography

Procedure-related events during the procedure, during the index hospitalization, and during the 45-day post-discharge observational period were counted and compared between those with and without hemodialysis. Of note, acute kidney injury was defined as an increase in serum creatinine level > 0.3 mg/dL per 48 h, 1.5-fold increase in serum creatinine level, or urine volume < 0.5 mL/kg/h for 6 h [12]. Device-related thrombus or residual peri-device flow > 5 mm in width were surveyed by transesophageal echocardiography (Fig. 3).

### Statistical analysis

Continuous variables were expressed as mean and standard deviation and compared between the two groups (i.e., hemodialysis group versus non-hemodialysis group) using unpaired t-test. Categorical variables were expressed as numbers and percentages and compared between the two groups using Fischer’s exact test. A value of *p* <0.05 was considered statistically significant. Statistical analyses were performed using SPSS Statistics 24 (SPSS Inc, Armonk, IL, USA).

## Results

### Baseline characteristics

118 NVAF patients were included. Median age was 79 (73, 84) years old and 81 (69%) were men (Table 1). On median, CHADS_2_ score was 3 (2, 4) points, CHADS_2_-VASc score was 5 (4, 6) points, and HAS-BLEAD score was 3 (2, 3) points.

**Table 1:** Baseline characteristics

	Total (*N* = 118)	Hemodialysis (*N* = 25)	Non-hemodialysis (*N* = 93)	*p* value
Demographics				
Age, years	79 (73, 84)	73 (71, 79)	80 (75, 84)	< 0.001*
Men	81 (69%)	20 (80%)	61 (66%)	0.13
Body mass index, kg/m^2^	23.3 (20.6, 25.5)	21.6 (20.0, 24.4)	23.4 (21.0, 25.6)	0.22
Vital signs				
Systolic blood pressure, mmHg	120 (105, 135)	125 (111, 136)	117 (104, 132)	0.069
Diastolic blood pressure, mmHg	67 (58, 79)	65 (55, 72)	68 (58, 79)	0.12
Pulse rate, bpm	65 (59, 74)	66 (58, 75)	68 (59, 74)	0.89
Comorbidity				
Heart failure	73 (62%)	9 (36%)	64 (69%)	0.003*
Hypertension	87 (74%)	16 (64%)	71 (76%)	0.025*
Dyslipidemia	65 (55%)	18 (72%)	47 (51%)	0.044*
Diabetes mellitus	38 (32%)	12 (48%)	26 (28%)	0.05
Ischemic heart disease	51 (43%)	14 (56%)	37 (40%)	0.11
Peripheral artery disease	11 (9%)	6 (24%)	5 (5%)	0.011*
Carotid artery stenosis	6 (5%)	3 (12%)	3 (3%)	0.076
Hepatic disease	5 (4%)	0	5 (5%)	0.24
Chronic obstructive pulmonary disease	3 (3%)	0	3 (3%)	0.49
History of major bleeding	59 (50%)	7 (28%)	52 (56%)	0.013*
Increased risk of fall	28 (24%)	4 (16%)	24 (26%)	0.23
History of ischemic stroke	47 (40%)	15 (60%)	32 (34%)	0.019*
History of hemorrhagic stroke	12 (10%)	3 (12%)	9 (9%)	0.48
History of transient ischemic attach	8 (7%)	1 (4%)	7 (7%)	0.53
Laboratory data				
Hemoglobin, g/dL	12.1 (10.8, 13.6)	11.3 (10.8, 12.1)	12.5 (10.9, 13.9)	0.017*
Serum albumin, g/dL	3.8 (3.5, 4.1)	3.6 (3.4, 3.9)	3.9 (3.6, 4.1)	0.010*
Estimated glomerular filtration ratio, mL/min/1.73m^2^	42 (24, 57)	5 (5, 7)	48 (35, 61)	< 0.001*
Plasma B-type natriuretic peptide, pg/mL	169 (82, 288)	394 (260, 745)	132 (66, 202)	< 0.001*
Prothrombin time with international normalized ratio	1.21 (1.04, 1.47)	1.41 (1.18, 1.86)	1.17 (1.01, 1.41)	0.004*
Echocardiography data				
Left ventricular end-diastolic diameter, mm	48 (44, 52)	52 (47, 55)	47 (43, 50)	0.77
Left ventricular ejection fraction, %	61 (52, 68)	64 (46, 75)	63 (57, 69)	0.76
Left atrial volume index, mL/m^2^	60 (44, 82)	45 (35, 57)	60 (41, 82)	0.17
Moderate or greater mitral regurgitation	12 (10%)	0	12 (13%)	0.058
Moderate or greater tricuspid regurgitation	17 (14%)	0	17 (18%)	0.021*
Mild or greater spontaneous contrast	64 (54%)	12 (48%)	52 (56%)	0.32
Score				
CHADS_2_ score	3 (2, 4)	3 (2, 4)	3 (2, 4)	0.98
CHADS_2_-VASc score	5 (4, 6)	5 (4, 6)	5 (4, 6)	0.66
HAS-BLEAD score	3 (2, 3)	4 (3, 5)	3 (2, 3)	< 0.001*
Clinical frailty scale	3 (3, 4)	3 (3, 4)	3 (3, 4)	0.87
Modified Rankin scale	0 (0, 1)	1 (0, 2)	0 (0, 1)	0.67

*p < 0.05. Continuous variables are stated as median and interquartile and compared between the groups using Mann–Whitney *U* test. Categorical variables are stated as number and percentage and compared between the groups using Fischer’s exact test

Of them, 25 patients were dependent on hemodialysis and other 93 patients were assigned to the non-hemodialysis group (Fig. 2). Hemodialysis patients were younger and had a lower incidence of major bleeding history and higher incidence of ischemic stroke history compared with the non-hemodialysis group (*p* < 0.05 for all). CHADS_2_ score and CHADS_2_-VASc score were not significantly different between the two groups, whereas the hemodialysis group had a higher HAS-BLEAD score (*p* < 0.001). Eighty patients received transesophageal echocardiography, which demonstrated no significant differences in the LAA parameters between the two groups (*p* > 0.05 for all; Table 2).

**Table 2:** Baseline transesophageal echocardiography data

	Total (*N* = 80)	Hemodialysis (*N* = 22)	Non-hemodialysis (*N* = 58)	*p* value
LAA ostium diameter				
LAA ostium diameter at 0 degree, mm	21 (19, 24)	21 (17, 24)	22 (20, 23)	0.61
LAA ostium diameter at 45 degrees, mm	20 (17, 22)	19 (15, 21)	20 (18, 22)	0.16
LAA ostium diameter at 90 degrees, mm	20 (19, 23)	20 (17, 24)	21 (19, 23)	0.65
LAA ostium diameter at 135 degrees, mm	22 (20, 26)	22 (19, 27)	22 (21, 26)	0.55
LAA depth				
LAA depth at 0 degree, mm	21 (15, 29)	29 (17, 33)	20 (13, 24)	0.22
LAA depth at 45 degrees, mm	27 (18, 33)	31 (23, 35)	25 (18, 31)	0.19
LAA depth at 90 degrees, mm	26 (17, 31)	27 (24, 35)	21 (17, 30)	0.13
LAA depth at 135 degrees, mm	24 (20, 30)	29 (20, 35)	24 (19, 28)	0.085
Flow velocity				
LAA filling flow velocity, m/sec	33 (20, 49)	45 (19, 57)	29 (20, 40)	0.20
LAA emptying flow velocity, m/sec	25 (17, 45)	43 (16, 54)	22 (18, 39)	0.12
PV flow velocity of S wave, m/sec	30 (17, 42)	39 (16, 49)	28 (18, 41)	0.22
PV flow velocity of D wave, m/sec	33 (21, 41)	31 (21, 41)	36 (21, 42)	0.81

*LAA* left atrial appendage, *PV* pulmonary vein

Continuous variables are stated as median and interquartile and compared between the groups using Mann–Whitney *U* test

### LAAC procedure

The procedure success rate was 99%: the procedure was unsuccessful in a patient in the non-hemodialysis group due to the inappropriately larger size of LAA (Table 3). The procedure time was longer and the contrast volume was higher in the hemodialysis group (*p* < 0.05 for both). The incidences of multiple device use and partial recapture were not significantly different between the two groups (*p* > 0.05 for both).

**Table 3:** Procedure data

	Total (*N* = 118)	Hemodialysis (*N* = 25)	Non-hemodialysis (*N* = 93)	*p* value
Procedure success	117 (99%)	25 (100%)	92 (99%)	0.60
Procedural parameters				
Anesthesia time, min	117 (101, 135)	126 (118, 161)	115 (100, 128)	0.002*
Procedural time, min	55 (43, 70)	65 (56, 96)	51 (41, 64)	< 0.001*
Fluoroscopy duration, min	15 (10, 20)	17 (13, 21)	14 (10, 18)	0.022*
Contrast volume, mL	65 (50, 80)	75 (60, 95)	60 (50, 75)	0.003*
Device data				
Device type				
WATCHMAN 2.5	48 (41%)	17 (68%)	31 (33%)	0.007*
WATCHMAN FLX	69 (58%)	8 (32%)	61 (66%)	0.007*
Device size, cm	31 (27, 33)	33 (27, 33)	31 (27, 33)	0.44
Implantation data				
Multiple device use	12 (10%)	5 (20%)	7 (8%)	0.067
Partial recapture	63 (53%)	15 (60%)	48 (52%)	0.60

*p < 0.05. Continuous variables are stated as median and interquartile and compared between the groups using Mann–Whitney *U* test. Categorical variables are stated as number and percentage and compared between the groups using Fischer’s exact test

During the index hospitalization, there were no major complications including access site events, acute kidney injury, pericardial effusion, device embolization, infectious endocarditis, and stroke (Table 4). One hemodialysis patient had bleeding event due to suspected gastrointestinal bleeding, which improved by discontinuing warfarin without blood transfusion. The in-hospital duration was not significantly different between the two groups (*p* = 0.76). All patients could be discharged alive.

**Table 4:** Peri-procedural and in-hospital data

	Total (*N* = 118)	Hemodialysis (*N* = 25)	Non-hemodialysis (*N* = 92)	*p* value
In-hospital duration, days	4 (4, 5)	4 (4, 5)	4 (4, 5)	0.81
Access site-related complication	0	0	0	–
Transesophageal echocardiography-related complication	0	0	0	–
Pulmonary complication	0	0	0	–
Acute kidney injury	0	NA	0	–
Pericardial effusion	0	0	0	–
Device embolization	0	0	0	–
Atrial septal defect requiring closure	0	0	0	–
Infectious endocarditis	0	0	0	–
Stroke	0	0	0	–
Any bleeding	1 (1%)	1 (4%)	0	0.053
All-cause death	0	0	0	–

Continuous variables are stated as median and interquartile and compared between the groups using Mann–Whitney *U* test. Categorical variables are stated as number and percentage and compared between the groups using Fischer’s exact test

### Trends in medications

At baseline, more hemodialysis patients received antiplatelets than the non-hemodialysis group (*p* < 0.05; Table 5). All hemodialysis patients received warfarin, whereas most of the non-hemodialysis patients (91%) received DOAC.

**Table 5:** Trends in antiplatelets and anticoagulants prescription at baseline and following the procedures

	Total	Hemodialysis	Non-hemodialysis	*p* value
Baseline (*N* = 117)	(*N* = 117)	(*N* = 25)	(*N* = 92)	
Antiplatelet				0.007*
None	67 (57%)	8 (32%)	59 (63%)	–
SAPT	48 (41%)	15 (60%)	33 (35%)	–
DAPT	3 (3%)	2 (8%)	1 (1%)	–
Warfarin	32 (27%)	25 (100%)	8 (9%)	< 0.001*
DOAC	85 (72%)	0	85 (91%)	< 0.001*
Discharge (*N* = 117)	(*N* = 117)	(*N* = 25)	(*N* = 92)	
Antiplatelet				0.011*
None	24 (21%)	1 (4%)	23 (25%)	–
SAPT	93 (79%)	24 (96%)	69 (74%)	–
DAPT	0	0	0	–
Warfarin	33 (28%)	25 (100%)	8 (9%)	< 0.001*
DOAC	85 (72%)	0	85 (91%)	< 0.001*
45-day follow-up (*N* = 94)	(*N* = 94)	(*N* = 21)	(*N* = 73)	
Antiplatelet				0.05
None	18 (19%)	0	18 (25%)	–
SAPT	76 (81%)	21 (100%)	55 (75%)	–
DAPT	0	0	0	–
Warfarin	25 (27%)	20 (95%)	5 (7%)	< 0.001*
DOAC	68 (72%)	0	68 (93%)	< 0.001*

*SAPT* single antiplatelets, *DAPT* dual antiplatelets, *DOAC* direct oral anticoagulants

*p < 0.05. Categorical variables are stated as number and percentage and compared between the groups using Fischer’s exact test

As for the antiplatelets, most of the patients (79%) received single antiplatelets at the index discharge according to the institutional protocol, irrespective of the dependence on hemodialysis (Fig. 2). No patients received dual antiplatelets.

94 patients were followed until day 45. The proportion of prescriptions remained almost unchanged from the index discharge. In the hemodialysis patients, 20 (95%) received warfarin. In the non-hemodialysis patients, 5 (7%) received warfarin and others (68 [93%]) received DOAC.

### Short-term follow-up data

94 patients completed a 45-day follow-up (Table 6). Most of the device parameters, which were assessed using transesophageal echocardiography, were not statistically different between the two groups. Almost half of them had procedure-related residual atrial septum defect, irrespective of the dependence on hemodialysis (*p* = 0.63). No patients had right-to-left jet.

**Table 6:** 45-day follow-up data including transesophageal echocardiography data

	Hemodialysis (*N* = 21)	Non-hemodialysis (*N* = 73)	*p* value
Device maximum diameter			
Device maximum diameter at 0 degree, mm	27.7 (23.6, 29.0)	26.8 (24.2, 29.0)	0.94
Device maximum diameter at 45 degrees, mm	26.4 (22.3, 29.6)	26.1 (13.7, 27.9)	0.81
Device maximum diameter at 90 degrees, mm	27.5 (23.2, 30.0)	26.2 (24.4, 29.0)	0.25
Device maximum diameter at 145 degrees, mm	28.0 (23.2, 30.0)	26.2 (24.4, 29.0)	0.54
Device compression			
Device compression at 0 degree, mm	14.3 (12.1, 19.4)	11.1 (9.2, 15.1)	0.004*
Device compression at 45 degrees, mm	15.5 (12.7, 23.3)	13.3 (10.0, 18.4)	0.077
Device compression at 90 degrees, mm	16.4 (9.3, 18.1)	12.1 (9.7, 17.4)	0.46
Device compression at 145 degrees, mm	14.5 (10.0, 19.7)	12.3 (9.1, 15.0)	0.31
Peri-device leak > 5 mm	0	1 (1%)	0.59
Device protrusion			
Device protrusion at 0 degree, mm	6.0 (4.0, 7.0)	6.4 (4.0, 8.4)	0.53
Device protrusion at 45 degrees, mm	5.8 (5.0, 6.9)	6.0 (4.2, 7.5)	0.95
Device protrusion at 90 degrees, mm	8.0 (4.9, 9.7)	6.1 (4.5, 8.0)	0.24
Device protrusion at 145 degrees, mm	8.0 (6.1, 11.0)	6.6 (5.3, 8.4)	0.05
ASD			
ASD direction			0.63
None	7 (33%)	34 (47%)	–
Left to right	12 (57%)	35 (48%)	–
Right to left	0	0	–
Bi-direction	1 (5%)	4 (5%)	–
ASD diameter, mm	3.0 (1.5, 3.5)	3.0 (2.0, 3.0)	0.98
ASD pressure gradient, mmHg	6.3 (4.5, 12.0)	5.0 (3.1, 8.7)	0.37
Complications			
Pericardial effusion	0	0	–
Device-related thrombus	0	0	–
Deep device implantation	0	0	–
All-cause death	0	1 (1%)	0.88

*ASD* atrial septum defect

*p < 0.05. Continuous variables are stated as median and interquartile and compared between the groups using Mann–Whitney *U* test. Categorical variables are stated as number and percentage and compared between the groups using Fischer’s exact test

During the 45-day observational period, no patients had major complications including pericardial effusion and device-related thrombus. There were no deceased patients, except for a patient with cardiac amyloidosis in the non-hemodialysis group who died suddenly without any obvious reasons 5 days following the index discharge.

## Discussion

In this prospective study, we reported for the first time the short-term feasibility of LAAC in hemodialysis patients with high risks of thromboembolic events and bleedings.

### Safety and efficacy of LAAC in the non-hemodialysis patients

LAAC is an established alternative to anticoagulation therapy to prevent stroke events in patients with NVAF and high risk of bleedings [9]. LAAC has a non-inferiority in efficacy and safety to long-term warfarin therapy in this cohort. A recent 4-year observational study demonstrated comparable safety and efficacy of LAAC to DOAC therapy also in this cohort [13]. More feasibility has been achieved by the innovation of WATCHMAN FLX device over the conventional WATCHMAN 2.5 [14]. However, the strategy to prevent stroke in patients with hemodialysis and NVAF remains uncertain.

### Prevention of stroke in the hemodialysis patients

In non-hemodialysis patients, the existence of NVAF is a major trigger of cardiac stroke. The hemodialysis patients have multiple high-risk origins of thromboembolic strokes due to systemic atherosclerosis, whereas cardiac stroke is reported to be a major etiology in Japan [15].

The applicability of CHADS_2_ score, which is an established score to consider the risk of stroke in the general cohort, to the hemodialysis cohort remains unknown, given that the original study did not include them [16]. However, the risk of stroke increases in NVAF patients with renal impairment compared with those without renal impairment [17].

Anticoagulation therapy is an established strategy to prevent stroke in the general cohort with NVAF and high risk of stroke. In hemodialysis patients, all types of DOAC are contraindicated. The Japanese society for dialysis therapy guidelines states that warfarin therapy is, in principle, contraindicated [18]. Preventive effect of warfarin on stroke remains unknown. Instead, warfarin may be associated with the incremental incidence of major bleeding [8]. Warfarin might facilitate atherosclerosis, which was assessed using pulse wave velocity analyses [19].

Given all together, strategies other than anticoagulation would be required for those with hemodialysis to prevent stroke.

### LAAC for hemodialysis patients

One of the strategies to answer the above request is LAAC. Gotzmann and colleagues retrospectively analyzed 128 candidates of LAAC. Of them, mortality, bleeding incidence, and thromboembolic events incidence were not statistically different between 33 hemodialysis patients and others [20]. In another study, renal impairment was associated with higher in-hospital mortality, longer hospital stay, and a higher 30-day readmission rate [21].

This is the first study that demonstrated the short-term feasibility of LAAC in Japanese hemodialysis patients. According to the recommended standard regimen for post-LAAC 45 days consisting of the combination of DOAC and low-dose aspirin [22], we administered DOAC (for non-hemodialysis patients) or warfarin (for hemodialysis patients) and low-dose aspirin and/or thienopyridine P2Y12 receptor antagonist. We adjusted the dose of warfarin with a target range of international normalized ratio between 1.5 and 2.0, according to the recommendation of guidelines.

The incidences of peri-procedural complications were not significantly different between those with and without hemodialysis. There was no device-related thrombosis during the 45-day follow-up period among all cohort. Only a patient with hemodialysis required termination of warfarin during a 45-day follow-up period. The patient had a slight progression of anemia due to asymptomatic interstitial bleeding, which was ameliorated without blood transinfusion.

LAAC might be a feasible strategy also in hemodialysis patients. Short-term warfarin therapy following LAAC would be safe in this cohort. Further studies are warranted to validate the long-term feasibility and implication of LAAC in this cohort.

### Limitations

This study was conducted in a single center using a small sample size. Statistical non-significance does not guarantee similarity. There are several differences in background and procedure-related parameters, including the incidence of major bleeding history and HAS-BLEAD score as well as procedure time between the two groups. More patients with hemodialysis received LAAC due to high HAS-BLEAD score rather than a history of major bleeding. Initial recommendations to receive LAAC were dominantly performed by cardiologists. Thus, there might be selection bias. We cannot ignore the impact of two different devices (2.5 and FLX) and the learning curve of the operators. Of note, hemodialysis patients trended to receive WATCHMAN 2.5 rather than FLX. Post-procedural medication regimens were different between the two groups. Such a difference might have affected clinical outcomes. We observed just 45 days following LAAC and a longer observational study is the next concern.

## Conclusions

LAAC seems to be feasible in hemodialysis patients with high risks of thromboembolic events and bleedings.

## Data Availability

Data are available from the corresponding authors upon reasonable requests.
